# Native Cultivable Bacteria from the Blueberry Microbiome as Novel Potential Biocontrol Agents

**DOI:** 10.3390/microorganisms10050969

**Published:** 2022-05-05

**Authors:** Florencia Isabel Chacón, Pedro Eugenio Sineli, Flavia Ivana Mansilla, Martina Maria Pereyra, Mariana Andrea Diaz, Sabrina Inés Volentini, Anja Poehlein, Friedhelm Meinhardt, Rolf Daniel, Julián Rafael Dib

**Affiliations:** 1 Pilot Plant for Microbiological Industrial Processes, National Scientific and Technical Research Council, San Miguel de Tucumán 4000, Tucumán, Argentina; florenciac@conicet.gov.ar (F.I.C.); pedro.sineli@conicet.gov.ar (P.E.S.); flavia.m@conicet.gov.ar (F.I.M.); mmpereyra@conicet.gov.ar (M.M.P.); marianadiaz@conicet.gov.ar (M.A.D.); 2 Institute Superior of Biological Research (INSIBIO), CONICET-UNT and Institute of Biological Chemistry “Dr. Bernabé Bloj”, Faculty of Biochemistry, Chemistry and Pharmacy, National University of Tucumán (UNT), San Miguel de Tucumán 4000, Tucumán, Argentina; sivolentini@conicet.gov.ar; 3 Genomic and Applied Microbiology & Göttingen Genomics Laboratory, Institute of Microbiology and Genetics, Georg-August University of Göttingen, 37077 Göttingen, Germany; apoehle3@gwdg.de; 4 Institute for Molecular Microbiology and Biotechnology, Westfälische Wilhelms University of Münster, 48149 Münster, Germany; meinhar@uni-muenster.de; 5 Institute of Microbiology, Faculty of Biochemistry, Chemistry and Pharmacy, National University of Tucumán, San Miguel de Tucumán 4000, Tucumán, Argentina

**Keywords:** blueberry, biocontrol, *Botrytis*, *Alternaria*, bacteria, *Bacillus*, *Asaia*

## Abstract

Blueberry production is affected by fungal postharvest pathogens, including *Botrytis cinerea* and *Alternaria alternata*, the causative agents of gray mold disease and Alternaria rot, respectively. Biocontrol agents adapted to blueberries and local environments are not known to date. Here, we report on the search for and the identification of cultivable blueberry epiphytic bacteria with the potential to combat the aforementioned fungi. Native, blueberry-borne bacterial strains were isolated from a plantation in Tucumán, Argentina and classified based on 16S rRNA gene sequences. Antagonistic activities directed at *B. cinerea* and *A. alternata* were studied in vitro and in vivo. The 22 bacterial strains obtained could be attributed to eleven different genera: *Rosenbergiella*, *Fictibacillus*, *Bacillus*, *Pseudomonas*, *Microbacterium*, *Asaia*, *Acinetobacter*, *Curtobacterium*, *Serratia*, *Sphingomonas* and *Xylophilus*. Three strains displaying antagonistic impacts on the fungal pathogens were identified as *Bacillus velezensis* (BA3 and BA4) and *Asaia spathodeae* (BMEF1). These strains are candidates for biological control agents of local blueberry production and might provide a basis for the development of eco-friendly, sustainable alternatives to synthetic pesticides.

## 1. Introduction

Blueberry (*Vaccinium corymbosum* L.), a plant belonging to the *Ericaceae* [1], has recently gained great commercial significance, which is not only due to the utile nutritional properties of the berries, but also—and at least equally importantly—their proven beneficial health effects. Ingredients such as polyphenols, anthocyanins, and phenolic acids account for the latter. Their health-promoting effects include prevention of diabetes, hyperlipidemia, hypertension, neurodegeneration, obesity, and osteoporosis. In addition, anti-inflammatory, antioxidant, and anticarcinogenic properties have been described [2,3,4,5].

In Argentina, production substantially increased within the last decade; nowadays, it is no. 7 worldwide with respect to blueberry harvest. The annual production of 18,000 tn is almost totally (95%) exported, especially to the United States, the Netherlands, and Germany [6,7].

*Botrytis cinerea* and *Alternaria alternata*, the causal agents of gray mold and *Alternaria* rot, respectively, are the most commercially significant postharvest fungal pathogens responsible for crop losses of fruits [8,9,10]. The *B. cinerea* infection is typically characterized by a soft rot brought about by water soaking and, finally, the collapse of tissues, rapidly followed by a massive formation of gray conidia on fruit surfaces [11,12]. *A. alternata*-infested berries display soft tissues along with a whitish, airy mycelium on the fruit surface; after two to three days, the hyphae fatten up and become olive green, brown, or black [13,14,15].

Countermeasures aimed at prevention of postharvest fungal fruit decay traditionally include fungicides such as anilinopyrimidines, phenylpyrrol, hydroxyanilides, and carboximides [16,17,18]. In addition to a possible upcoming ban on artificially synthesized fungicides, the extensive use of chemical compounds in agriculture entails a number of disadvantages, such as the increasing public concern of their toxicity along with severe negative environmental impacts, the emergence of resistant phytopathogenic strains, and expectable trade barriers. Thus, emphasis was placed on the development of novel, sustainable, eco-friendly, and safe biocontrol technologies [19,20,21].

Biological control is one of the most potent alternatives to synthetic fungicides, especially as the implementation of native epiphytic microorganisms to combat postharvest pathogens has proved successful [22]. Organisms colonizing the respective ecological niche not only show the adaptive advantages of candidate microbes, but also increase public acceptance [23]. To date, several bacteria-based biocontrol agents are commercially available, such as BioSave^®^ or Serenade^®^; both biocontrol formulations employ prokaryotes, i.e., *Pseudomonas syringae* ESC-10 and *Bacillus subtilis* QST-713, respectively. Although—amongst others (such as strawberry and grape)—the above microorganisms also bear the potential to protect blueberries [24], native specimens adapted to the corresponding local fruit environment and, at the same time, displaying potential biocontrol capacities are not yet known.

The aim of this study was the isolation and evaluation of epiphytic bacteria from the blueberry microbiome with the potential to combat the most harmful blueberry postharvest fungal phytopathogens.

## 2. Materials and Methods

### 2.1. Sample Collection

Fruits and flowers belonging to Snowchaser blueberry cultivars were collected from plantations belonging to the Tierra de Arándanos company in Monteros, Tucumán, Argentina. Samples without any preharvest treatment with synthetic products (herbicides, pesticides) were transported to the laboratory in sterile bags using a cooler box. Permission to conduct these studies was granted by the local authority: “Dirección de Flora, Fauna Silvestre y Suelos” (Department of Flora, Fauna and Soils), Tucumán, Resolution N° 20-2022 (DFFSyS).

### 2.2. Cultures and Preparation of Conidial Suspensions of the Pathogens

Phytopathogenic strains of *B. cinerea* ISIB-MMA/F-Bc01-S and *A. alternata* ISIB-MMA/F-Alsp19-S were obtained from the strain collection of INSIBIO-CONICET (Higher Institute of Biological Research) in Tucumán, Argentina.

Conidial suspensions were prepared by collecting conidiospores from a 10-day culture grown on PDA medium (4 g L^−1^ potato extract, 20 g L^−1^ glucose, 15 g L^−1^ agar, pH 5.6) at 25 °C. Standard saline solution containing 0.1% Tween 80 was evenly spread on the surface of the mycelia and gently scraped employing a sterile loop to ensure conidial harvesting. The obtained conidial suspensions were adjusted to a concentration of 10^6^ conidia mL^−1^ using a Neubauer’s counting chamber.

### 2.3. Isolation of Bacteria

Flower and fruit (20 g) samples were placed in sterile flasks containing 100 mL of the above saline solution with 0.1% Tween 80. The flasks were vigorously shaken (200 rpm) for 30 min in an orbital shaker (Biomint, Buenos Aires, Argentina). Subsequently, 100 µL samples were ten-fold serially diluted and plated on two different isolation media—(i) LB-agar medium (5 g L^−1^ yeast extract, 10 g L^−1^ tryptone, 10 g L^−1^ NaCl, 15 g L^−1^ agar, pH 7) and (ii) LBAr medium (500 mL L^−1^ blueberry juice, 0.25 g L^−1^ yeast extract, 5 g L^−1^ tryptone, 5 g L^−1^ NaCl, 15 g L^−1^ agar)—both of which were supplemented with 50 mg L^−1^ cycloheximide to prevent fungal growth. The plates were incubated at 25 °C for up to 72 h. Single colonies showing different macroscopic appearances were isolated and the bacterial morphology determined by microscopy and Gram staining. Pure cultures of each bacterial isolate were conserved in LB-medium containing 20% glycerol and kept at −80 °C for long-term storage.

### 2.4. Assaying the In Vitro Antagonistic Activity against Blueberry Pathogenic Fungi

Inhibitory effects on the mycelial growth of *B. cinerea* and *A. alternata* of all the isolates were primarily assessed in vitro, applying a slightly modified dual culture assay [25]. Briefly, 100 µL of the conidial suspension (10^6^ conidia mL^−1^) was seeded on PDA plates (90 mm diameter); subsequently, 5 µL of the respective bacterial suspension (10^8^ to 10^9^ CFU mL^−1^) grown to the late exponential phase in LB was spotted on equidistant sites of the plate. Each experimental setting was done in triplicate. The plates were evaluated in daily intervals. Samples causing growth inhibition were classified as antagonists and selected for further analysis.

The selected bacterial candidates were tested individually in order to quantify the inhibition potential with respect to the mycelial growth of the phytopathogens by applying the more accurate parallel streak method according to the protocol given in De Lima et al. (2012) [26] with some modifications: 5 µL samples of the phytopathogen conidial suspension were spotted in the center of a PDA plate (90 mm diameter). A loopful of the bacterial isolate to be tested was streaked on each of two sites adjacent to the phytopathogen, at a distance of 20 mm to the plate edge. The controls consisted of PDA plates solely inoculated with the fungus. Petri dishes were incubated at 25 °C until the fungal growth of controls reached the plate edge, which took 10 days in the case of *B. cinerea* and 7 days for *A. alternata*. After incubation, the radial growth was measured, and the inhibition percentage was calculated as the percentage of colony radial growth decrease compared to the control.

### 2.5. In Vivo Biocontrol Efficiency

The efficiency of the selected bacterial isolates in blueberry protection against *B. cinerea* was assessed as described by Olmedo (2017) [27] with some modifications. Bacterial cultures were grown in LB broth for 24 h at 25 °C and 180 rpm and bacterial suspensions (10^8^ to 10^9^ CFU mL^−1^) were directly used to treat fruits. Blueberries (3 replicates of 10 berries each) were wounded in the equatorial zone with a sanitized needle and disposed in clean plastics trays. Then, the fruits were treated with the tested bacterial suspension by aspersion (for 10 fruits, 3 mL of bacterial suspension was used) and were incubated for 24 h at 25 °C before being infected by the fungal phytopathogen. A fungal conidial suspension (10^6^ conidia mL^−1^) was used to artificially infect the fruits by spraying. These infected berries were further incubated in plastic trays for 7 days at 25 °C and 95% relative humidity. The controls consisted of wounded blueberries treated solely with LB medium and *B. cinerea*. The protection efficiency was evaluated and calculated as the number of healthy berries per treatment using the following equation:

$$\mathrm{Protection}\;\mathrm{efficiency}\;\left(\%\right)=\left(\frac{\mathrm{number}\;\mathrm{of}\;\mathrm{healthy}\;\mathrm{fruits}}{\mathrm{total}\;\mathrm{number}\;\mathrm{of}\;\mathrm{fruits}}\right)\times 100$$

### 2.6. Molecular Identification of Bacterial Isolates

The identification of isolates from blueberries was carried out by sequencing a fragment of the 16S rRNA gene. Thus, the gene of 16S rRNA, which has approximately 1500 bp, was amplified for each isolate [28]. The strains were grown in 15 mL LB medium (10 g L^−1^ tryptone, 10 g L^−1^ NaCl, 5 g L^−1^ yeast extract) at 25 °C and 180 rpm in an orbital shaker (Biomint, Buenos Aires, Argentina) for 48 h. The cells were harvested by centrifugation (10,000× *g* for 5 min), and DNA extractions were carried out using a genomic DNA extraction kit (QIAamp DNA Kit, Qiagen, Hilden, Germany) according to the manufacturer´s protocol. Such DNA samples were used as the template for PCR amplification employing universal primers 8F (5′-AGAGTTTGATCCTGGC-3′) and 1504R (5′- TACCTTGTTACGACTT- 3′) [29]. The sizes of all amplification products were confirmed by electrophoresis on 1% agarose gels. The purified fragments were sequenced using the Sanger sequencing platform provided by Microsynth Seqlab (Göttingen, Germany). The identity of bacterial isolates was assigned by comparing the obtained DNA sequences with those of type strains available from the GenBank NCBI (National Center for Biotechnology Information, Bethesda (MD), USA) database applying BLAST (Basic Local Alignment Search Tool, Bethesda (MD), USA, https://blast.ncbi.nlm.nih.gov/Blast.cgi, accessed on 10 January 2022) [30].

### 2.7. Sequence Data Availability

The 16S rRNA gene sequences of the bacterial isolates are available from GenBank-NCBI under the nucleotide accession numbers: OL672313-OL672331.

### 2.8. Statistical Analysis

Data were analyzed by ANOVA, and mean values were compared with Tukey’s test at the 5% significance level. The InfoStat/L software, v2020, Grupo InfoStat, FCA, National University of Cordoba, Argentina [31] was used for the statistical analysis.

## 3. Results

### 3.1. Isolation of Bacteria from Blueberry

Initially, 22 different bacterial strains were isolated, of which 18 were isolated from LB agar, and 4 originated from the enriched LBAr medium. However, from the latter, only one isolate was able to grow in LB liquid medium and could be included in the continuing assays. Hence, 19 bacterial strains were further analyzed, of which 12 originated from blueberry flowers and 7 from berries (Table 1).

### 3.2. Molecular Identification

Representatives of three different phyla were disclosed among isolates: *Proteobacteria* (11), *Firmicutes* (5), and *Actinobacteria* (3). Altogether, members of eleven different genera were identified: *Rosenbergiella* (4), *Fictibacillus* (3), *Bacillus* (2), *Pseudomonas* (2), *Microbacterium* (2), *Asaia* (1), *Acinetobacter* (1), *Curtobacterium* (1), *Serratia* (1), *Sphingomonas* (1), and *Xylophilus* (1) (Table 1).

### 3.3. In Vitro Antagonism against Blueberry Pathogenic Fungi

In our preliminary tests (dual culture assays outlined in Material and Methods), eight strains displayed antagonistic activities against *B. cinerea*, whereas six strains obstructed the growth of *A. alternata*. Three isolates, i.e., BA3, BA4, and BMEF1, depicted retarding activities directed at both fungi (Table 2).

**Figure 1:**
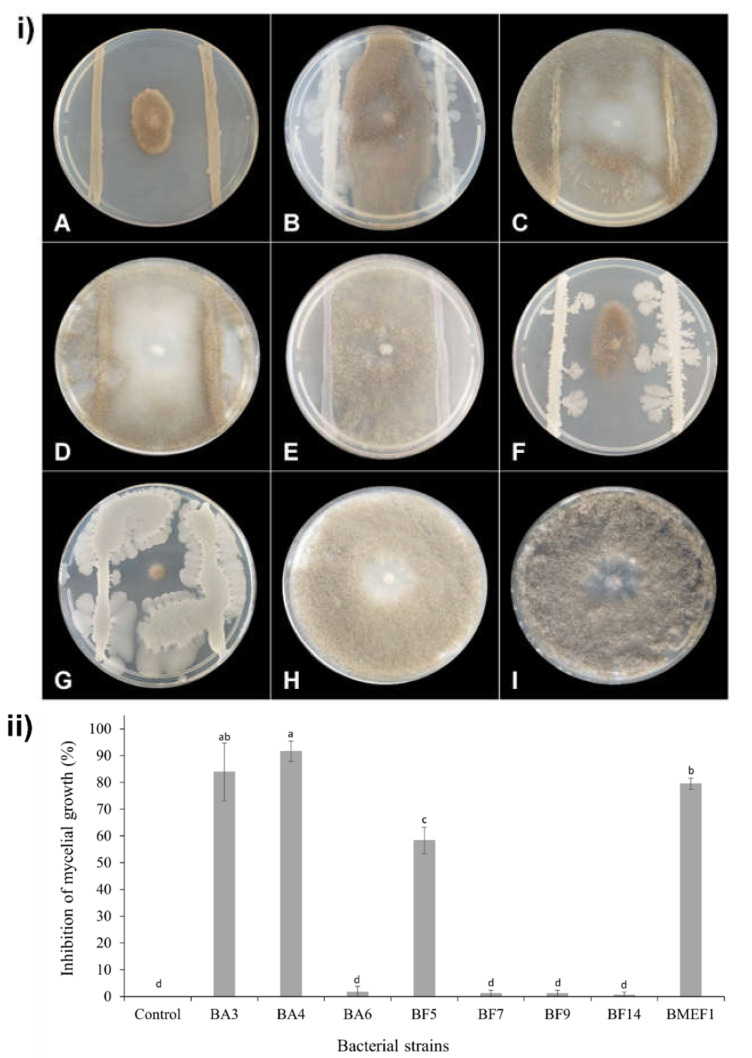
In vitro inhibitory activities of isolated bacteria against *B. cinerea* on PDA medium after 10 days of incubation at 25 °C. (**i**) Plates inoculated with the pathogen and bacterial strains: *Asaia spathodeae* BMEF1 (A), *Pseudomonas tremae* BF5 (B), *Microbacterium testaceum* BF7 (C), *Curtobacterium pusillum* BF9 (D), *Serratia marcescens* BF14 (E), *Bacillus velezensis* BA3 (F), *B. velezensis* BA4 (G), *Sphingomonas zeae* BA6 (H), respectively. (I) Control plate inoculated only with *B. cinerea*. (**ii**) Growth inhibition of *B. cinerea* confronted with bacterial isolates in dual culture assay. Different letters above the bars indicate significant differences according to Tukey’s test (*p* < 0.05).

**Figure 2:**
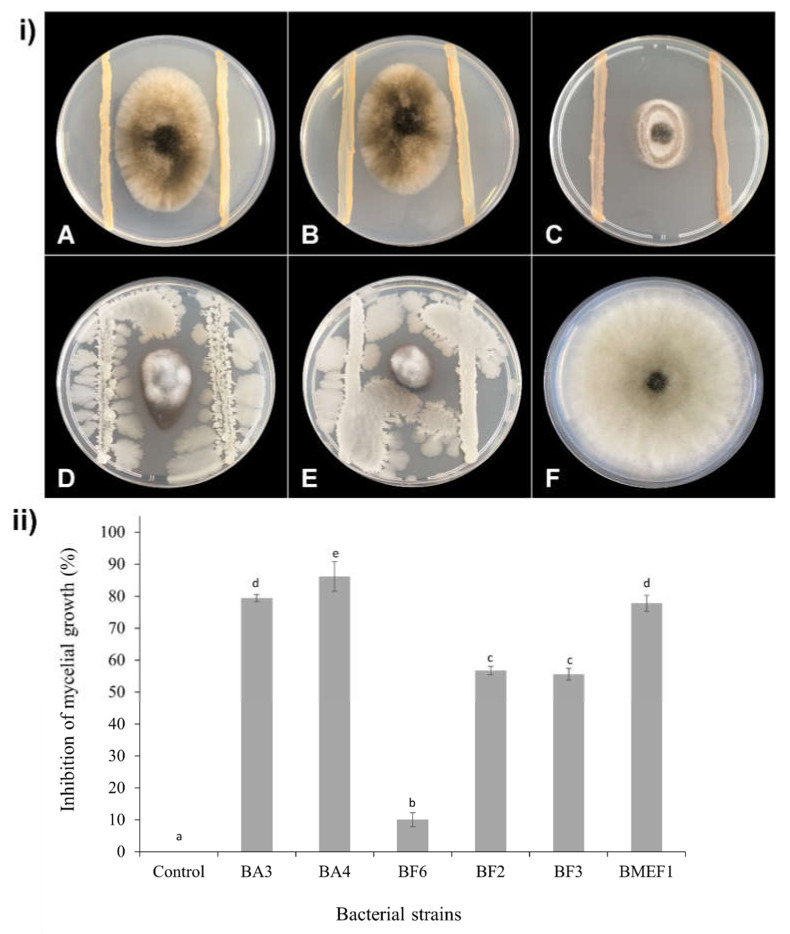
(**i**) In vitro inhibitory activities of isolated bacteria against *A. alternata* on PDA medium after 7 days of incubation at 25 °C.Plates inoculated with the pathogen and bacterial strains: *Rosenbergiella epipactidis* BF2 (A), *Rosenbergiella epipactidis* BF3 (B), *A. spathodeae* BMEF1 (C), *B. velezensis* BA3 (D), *B. velezensis* BA4 (E), respectively. (F) Control plate inoculated only with the fungus. (**ii**) Growth inhibition of *A. alternata* confronted with bacterial isolates in dual culture assay. Different letters above the bars indicate significant differences according to Tukey’s test (*p* < 0.05).

The eleven strains displaying any inhibiting activities in the above assay (BA3, BA4, BA6, BF2, BF3, BF5, BF6, BF7, BF9, BF14, and BMEF1) were subjected to the parallel streak method to quantify fungal growth inhibition by measuring mycelial growth after 10 and 7 days for *B. cinerea* (Figure 1) and *A. alternata* (Figure 2), respectively. From the eight strains inhibiting *B. cinerea*, BA3 and BA4 caused a growth impairment of over 80%; BMEF1 and BF5 caused an inhibition between 50 and 80%; BA6, BF7, BF9, and BF14 showed a lesser inhibition (10%). From the six strains harming *A. alternata*, BA4 inhibited growth by over 80%; strains BA3, BMEF1, BF2, and BF3 produced a growth impairment from 50 to 80%; BF6 revealed 10% inhibition.

### 3.4. In Vivo Biocontrol Capacities

**Figure 3:**
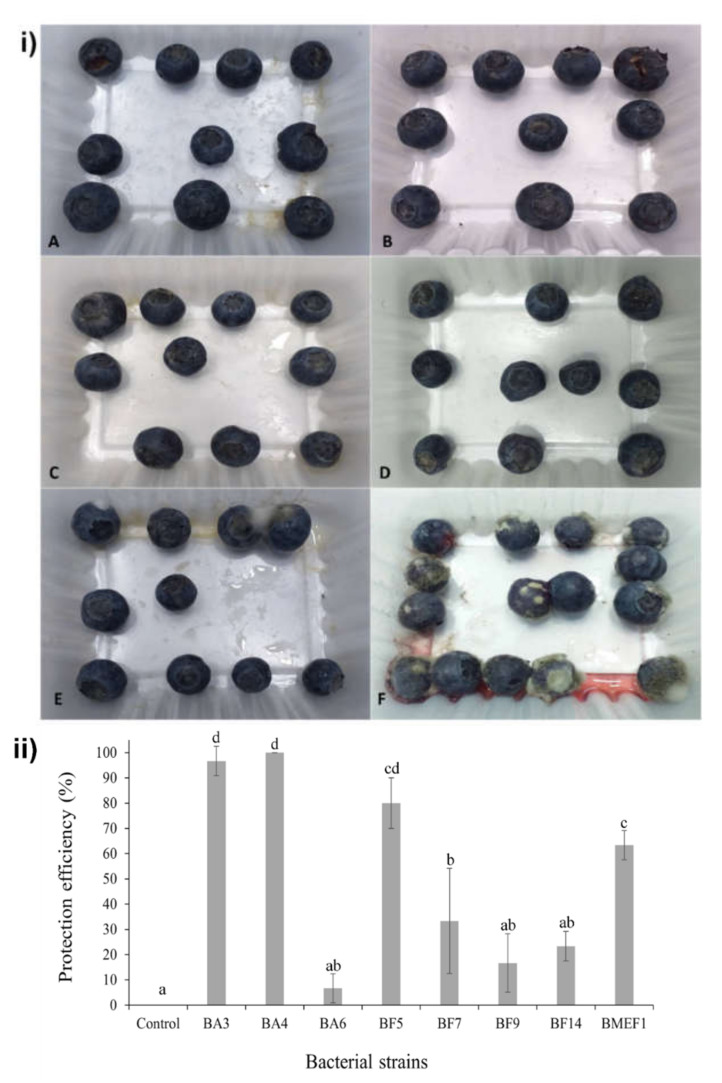
In vivo protection efficiency assay. (**i**) Blueberries artificially infected with *B. cinerea* after 7 days incubation at 25 °C. Fruits were pretreated as described in Material and Methods with strains *B. velezensis* BA4 (A); *B. velezensis* BA3 (B), *P. tremae* BF5 (C), *A. spathodeae* BMEF1 (D), and *M. testaceum BF7* (E), respectively. Control: fruits only inoculated with the pathogen (F). (**ii**) Protection efficiency of bacteria against *B. cinerea* in the in vivo assay. The 8 isolates producing *B. cinerea* growth inhibition in vitro (Table 2) were evaluated for their capacity to inhibit the fungus on blueberry fruits after 7 days of incubation at 25 °C. Different letters above the bars indicate significant differences by Tukey’s test (*p* < 0.05).

In vivo assays targeted at *B. cinerea* were conducted using blueberry fruits (as outlined in Material and Methods) using the eight selected isolates shown above (Table 2). After 7 days of incubation at 25 °C, *B. velezensis* BA3, *B. velezensis* BA4, *A*. *spathodeae* BMEF1, and *P. tremae* BF5 produced protection efficiencies greater than 60% (Figure 3). The strains *S. zeae* BA6, *M. testaceum* BF7, *C. pusillum* BF9, and *S. marcescens* BF14 showed no significant effects in protecting the fruits against the fungus.

## 4. Discussion

The lack of specific biocontrol agents facilitating the obviation of postharvest fungal infestations of blueberries, along with the drawbacks brought about by the use of synthetic fungicides, encouraged our study to find alternative biocontrol prospects. We looked for native bacteria from blueberries with the potential to exert antagonistic activities on the two most significant fungi with respect to crop losses, *B. cinerea* and *A. alternata*.

Since blueberry flowers and fruits are the plant organs most severely affected by the above fungal pathogens, we focused on biocontrol agents originating from the same environment in which they are destined to be finally applied, thereby widely ensuring both adaptation and survival [23,32]. In addition to non-supplemented medium (LB) a culture medium enriched with blueberry juice to mimic the natural environment of the fruit was employed (LBAr), but only one isolate could be further cultivated from the latter. Presumably, there are ingredients in the blueberry juice essential for some species that are lacking in the non-supplemented medium. Experiments to identify and characterize such substances are in progress.

To the best of our knowledge, this is the first report on the isolation and characterization of epiphytic bacteria with biocontrol potential from blueberry flowers and fruits.

The dual culture assay in our study again proved successful as a valuable qualitative approach to quickly assess antagonistic activities of microorganisms directed at fungal postharvest phytopathogens [25,33,34,35,36].

Paradigmatic testing of the protection efficiency of the different bacterial isolates against *B. cinerea* by in vivo application of both the bacterial strain and the fungus directly on the wounded fruits was carried out in a way that took into account a significant factor for commercialization. To date, most reports applied inoculation of the biocontrol agent directly at the wounded site [35,37,38,39,40]. Although being a quick and straightforward strategy, it is hardly compatible with conventional application conditions during fruit packaging. The spraying method applied in this study meets the producer´s requirements almost perfectly. From the sequence analysis of a part of the 16S rRNA gene, the three strains displaying the most persuasive antagonistic effects against both fungi were identified as *B. velezensis* (BA3 and BA4) and *A. spathodeae* (BMEF1). Inhibiting activities directed at different phytopathogens qualifies such candidates for the composition of a broad-spectrum biofungicide aimed at combating postharvest diseases. Studies on combined, possibly synergistic actions are in progress. The compelling antagonistic capacities of *B. velezensis* BA3 and BA4 agree with a number of literature reports focusing on biocontrol capabilities of species belonging to the genus *Bacillus*, which excel in their ability to produce a wide range of bioactive antimicrobial substances such as lipopeptides, antibiotics, enzymes, polyketides, and non-ribosomal proteins [36,41,42,43]. Since the members of the genus are characterized by their ability to produce heat, drought, and solvent-tolerant endospores, they are, with respect to durability, almost ideally suited for their inclusion in biocontrol formulations [44,45]. Previously, *B. velezensis* was a heterotypic synonym of *B*. *amyloliquefaciens*, based on DNA–DNA relatedness values. Currently, *B*. *velezensis* can be distinguished from B. *amyloliquefaciens* and *B. subtilis* based on multilocus sequence analysis [46].

Interestingly, there are rather recent reports presumably widening the range of the *B. velezensis* BA3 and BA4 disease-controlling efficacy and emphasizing their relevance as biocontrol agents: Kim et al. (2021) [47] reported on the activity of *B. velezensis* AK-0 against *Colletotrichum gloeosporoides*, the causal agent of apple bitter rot, while Palazzini et al. (2016) [48] demonstrated, in greenhouse and field trials, the ability of *B. velezensis* RC 218 to reduce the disease severity of the *Fusarium* head blight along with the diminution of deoxynivalenol, a disease-associated toxin. Moreover, *B. velezensis* NKG2 displayed in vitro antagonistic effects against several important fungal plant pathogens such as *B. cinerea*, *A. alternata*, *Fusarium graminareum*, *Fusarium oxysporum*, *Fulvia fulva*, and *Ustilaginosa virens* [49]. Thus, our results not only agree with the above reports on in vitro bioactivities of the species, but for the first time, antifungal *B. velezensis* strains were isolated from and tested against blueberry pathogens in vitro and in vivo.

*A. spathodeae* was, agreeing with the species designation, first isolated from flowers of *Spathodea campanulata*, the African tulip [50]. Studies on antagonistic activities are lacking to date. Thus, our results indicated that members of the species have antifungal activity and opened the avenue for application of BMEF1 as a biocontrol agent.

Although not as pronounced as for *A. spathodeae*, BMEF1 bioactivity against agriculturally important fungal species could also be attributed, for the first time, to strains of other, only rather recently discovered species, i.e., *Rosenbergiella epipactidis* [51] strains BF2, BF3, and BF6.

In conclusion, two native strains of *B. velezensis* originating from blueberry fruits and one strain of *A. spathodeae* from blueberry flowers displaying capacities to control the phytopathogenic fungi *Botrytis* and *Alternaria*, respectively, were isolated. These three strains are candidates for serving as biological control agents for local blueberry production. Future studies will focus on action mechanisms facilitating biological control by searching for bioactive compounds. In addition, the sequencing of the entire genome of the candidates will contribute to the identification and understanding of the genetic and physiological mechanisms involved in controlling effects on phytopathogenic fungi.

## Figures and Tables

**Table 1:** Identification of bacterial isolates from blueberries based on 16S rRNA gene sequencing.

Isolate	Source	Closest Related Species with Acc No. in Brackets and % Similarity	GenBank Accession No.	Sequence Length (bp)
BA2	Blueberry fruit	*Pseudomonas stutzeri* CCUG 11256T (NR_118798.1) 99.86%	OL672313	1401
BA3	Blueberry fruit	*Bacillus velezensis* strain FZB42 (NR_075005.2) 99.86%	OL672314	1407
BA4	Blueberry fruit	*Bacillus velezensis* strain FZB42 (NR_075005.2) 99.93%	OL672315	1397
BA6	Blueberry fruit	*Sphingomonas zeae* JM-791 (NR_136793.1) 98.89%	OL672316	1349
BA8	Blueberry fruit	*Fictibacillus nanhaiensis* strain JSM 082006 (NR_117524.1) 99.93%	OL672317	1424
BA9	Blueberry fruit	*Acinetobacter lwoffii* strain DSM 2403 (NR_026209.1) 99.85%	OL672318	1370
BA10	Blueberry fruit	*Fictibacillus phosphorivorans strain Ca7* (NR_118455.1) 99.93%	OL672319	1385
BF1	Blueberry flower	*Rosenbergiella epipactidis* strain 2.1A (NR_126303.1) 99.86%	OL672320	1380
BF2	Blueberry flower	*Rosenbergiella epipactidis* strain 2.1A (NR_126303.1) 99.78%	OL672321	1376
BF3	Blueberry flower	*Rosenbergiella epipactidis* strain 2.1A (NR_126303.1) 99.85%	OL672322	1372
BF5	Blueberry flower	*Pseudomonas tremae* strain TO1 (NR_025549.1) 99.78%	OL672323	1355
BF6	Blueberry flower	*Rosenbergiella epipactidis* strain 2.1A (NR_126303.1) 99.78%	OL672324	1373
BF7	Blueberry flower	*Microbacterium testaceum* strain DSM 20166 (NR_026163.1) 99.85%	OL672325	1320
BF8	Blueberry flower	*Xylophilus ampelinus* strain ATCC 33914 (NR_114461.1) 98.16%	OL672326	1358
BF9	Blueberry flower	*Curtobacterium pusillum* strain DSM 20527 (NR_042315.1) 99.48%	OL672327	1341
BF13	Blueberry flower	*Fictibacillus phosphorivorans* strain Ca7 (NR_118455.1) 99.93%	OL672328	1370
BF14	Blueberry flower	*Serratia marcescens strain* NBRC 102204 (NR_114043.1) 100%	OL672329	1310
BF15	Blueberry flower	*Microbacterium testaceum* strain DSM 20166 (NR_026163.1) 99.02%	OL672330	1328
BMEF1	Blueberry flower	*Asaia spathodeae* NBRC 105894 (NR_114292.1) 99.92%	OL672331	1300

**Table 2:** In vitro preliminary screening of bacterial isolates obtained by the dual culture assay with *B. cinerea* and *A. alternata* as the test organisms.

Bacterial Strain	Phytopathogen	
	*Botrytis cinerea*	*Alternaria alternata*
*Pseudomonas stutzeri* BA2	−	−
*Bacillus velezensis* BA3	+	+
*Bacillus velezensis* BA4	+	+
*Sphingomonas zeae* BA6	+	−
*Fictibacillus nanhaiensis* BA8	−	−
*Acinetobacter lwoffii* BA9	−	−
*Fictibacillus phosphorivorans* BA10	−	−
*Rosenbergiella epipactidis* BF1	−	−
*Rosenbergiella epipactidis* BF2	−	+
*Rosenbergiella epipactidis* BF3	−	+
*Pseudomonas tremae* BF5	+	−
*Rosenbergiella epipactidis* BF6	−	+
*Microbacterium testaceum* BF7	+	−
*Xylophilus ampelinus* BF8	−	−
*Curtobacterium pusillum* BF9	+	−
*Fictibacillus phosphorivorans* BF13	−	−
*Serratia marcescens* BF14	+	−
*Microbacterium testaceum* BF15	−	−
*Asaia spathodeae* BMEF1	+	+

Note:− = no inhibiting activity; + = inhibiting activity.

## Data Availability

Not applicable.
